# Salvage Stereotactic Body Radiation Therapy for Locally Recurrent Previously Irradiated Head and Neck Squamous Cell Carcinoma: An Analysis from the RSSearch® Registry

**DOI:** 10.7759/cureus.3237

**Published:** 2018-08-31

**Authors:** Hayden Ansinelli, Raj Singh, Dana L Sharma, Jan Jenkins, Joanne Davis, John A Vargo, Sanjeev Sharma

**Affiliations:** 1 Department of Radiation Oncology, University of Arizona College of Medicine, Tucson, USA; 2 Department of Radiation Oncology, Virginia Commonwealth University, Richmond, USA; 3 Department of Radiation Oncology, Marshall University Joan C. Edwards School of Medicine, Huntington, USA; 4 Clinical Programs, The Radiosurgery Society, San Mateo, USA; 5 Executive Director, The Radiosurgery Society, San Mateo, USA; 6 Department of Radiation Oncology, West Virginia University School of Medicine, Morgantown, USA; 7 Department of Radiation Oncology, St. Mary's Medical Center, Huntington, USA

**Keywords:** recurrent, squamous cell carcinoma, re-irradiation, dose escalation, stereotactic body radiotherapy, sbrt

## Abstract

Objectives

To report on overall survival (OS), local control (LC), dose-outcome relationships, and related toxicities following stereotactic body radiation therapy (SBRT) for locally recurrent, previously irradiated squamous cell carcinoma of the head and neck (rSCCHN).

Methods

We queried the prospectively-maintained RSSearch® Registry for patients with rSCCHN treated with five-fraction SBRT from January 2008 to November 2016. Patients with non-squamous cell histology, missing registry data regarding prior irradiation, those treated with less than five fractions of SBRT, and those treated with SBRT in primary or boost settings were excluded. LC and OS were estimated using the Kaplan-Meier method with comparisons between groups completed using log-rank t-tests and multivariable Cox regression. Logistic regression analyses were used to examine factors predictive of toxicity.

Results

Forty-five rSCCHN patients treated with SBRT delivered in five fractions at 12 radiotherapy centers were identified. Prescription doses ≥ 40 Gy were associated with higher one-year rates of OS, LC, and a higher likelihood of experiencing toxicities. Acute and late toxicity rates were low (22.2% and 15.6%, respectively) and were all Grade 1-2 with only one late Grade 3 esophagitis.

Conclusion

Salvage SBRT for rSCCHN resulted in outcomes comparable to prior single-institutional reports in a multi-institutional cohort across clinical settings with low toxicity, thus supporting more widespread adoption of SBRT with recommended doses ≥ 40 Gy.

## Introduction

Stereotactic body radiation therapy (SBRT) has emerged as a viable re-irradiation strategy for unresectable locally recurrent previously irradiated squamous cell carcinomas of the head and neck (rSCCHN), with recent multi-institutional comparative effectiveness analyses suggesting less acute toxicity and comparable outcomes when contrasting SBRT to intensity-modulated radiation therapy (IMRT) [1-2]. However, a wide range of dose selection exists across the SBRT literature, amidst the concern of increased toxicity rates with use of higher dose re-irradiation regimens [3].

The first Phase I dose-escalation trial using SBRT for rSCCHN reported a median overall survival (OS) of six months with no Grade 3/4 toxicities from dose tiers ranging from 25 Gy to 44 Gy [4]. This data was followed by reports which demonstrated that dose escalation to 50 Gy in five fractions was feasible with SBRT for rSCCHN [5]. Additionally, studies have shown that SBRT at a median dose of 40 Gy, administered with cetuximab, resulted in superior median survival compared to SBRT alone (24.5 vs. 14.8 months, respectively) and improved patient-reported quality of life [6-7]. 

Despite this data, multiple other studies have utilized a variety of dose regimens between 18 - 50 Gy in anywhere from one to six fractions, demonstrating median survival rates ranging from 6.7 to 13.6 months and one-year OS rates ranging from 38% to 58% [8-11]. Prospective Phase II studies of SBRT and cetuximab have similarly used variable doses of 36 - 50 Gy in five to six fractions [12-14]. As SBRT is pushed forward into the cooperative group setting in studies, such as the NRG KEYSTROKE trial (NCT03546582), the lack of consensus regarding dose selection across treatment settings has led to challenges in both clinical situations and trial design [15].

The RSSearch® Patient Registry is a recently developed multi-institutional international database designed to expedite the collection of clinical information for patients treated with stereotactic radiosurgery (SRS) or SBRT at community and academic radiotherapy centers. Since 2006, the registry has attained information regarding over 18,000 enrolled patients treated with SRS or SBRT. Previously, the registry has been a useful tool in the examination of outcomes in patients with various forms of cancer treated with SBRT, with results comparable to other investigational studies and research [16-18]. Thus, we aimed to use the RSSearch Patient Registry to compare toxicity, tumor control, and survival across a wide array of clinical practice settings to better guide dose selection. We hypothesized that higher dose selection is associated with improved local control and survival with no significant difference in toxicity.

## Materials and methods

The RSSearch Patient Registry encourages all centers utilizing SBRT as a treatment modality to voluntarily participate in the registry. Prior to participation in the registry, respective Institutional Review Board and Ethics Committee approval is required. Patients are asked for informed consent before having their respective information logged in the registry. No compensation is provided to either providers or patients for participating in RSSearch. Planning of SBRT plans and treatment delivery differed per respective institutional preferences.

The RSSearch Patient Registry was searched for patients with recurrent head and neck cancer (rHNC) of squamous cell histology treated with SBRT between January 2008 to November 2016. Inclusion criteria required that information was available regarding patient and lesion characteristics, treatment dose, and follow-up for survival analysis. Initial gross tumor volume (GTV) and local control (LC) were evaluated either by computed tomography (CT), magnetic resonance imaging (MRI), or positron-emission tomography (PET)/CT as per institutional preference. 

Statistical summaries of the relevant patient and lesion characteristics, in addition to radiotherapy planning, were performed with descriptive analyses. Possible factors impacting LC and OS were identified via the Kaplan-Meier method, with a comparison between groups completed using log-rank t-tests. Prognostic factors that were evaluated included prescription dose and fractionation schedule, tumor location, initial Karnofsky Performance Score (KPS), patient age, and GTV. When examining the benefit of dose escalation, doses of ≥ 40 Gy were compared against doses < 40 Gy, given prior reports documenting LC benefits with prescription doses of 40 Gy and above for five-fraction SBRT. To account for the study’s retrospective design and potential indication bias, a Cox multivariate analysis (MVA) was performed. The MVA was completed first utilizing a forced entry method of all analyzed variables, then as a sensitivity analysis due to the small number of patients relative to the number of variables by means of a parsimonious model formed using a forward selection of only variables significant on univariate analysis. 

The relationship between side-effect incidence and dose escalation was evaluated via logistic regression. Acute toxicities were defined as occurring within three months of the end of treatment and late toxicities as those occurring more than three months following treatment. KPS and pain scores (as measured by the visual analog score (VAS) on a scale of 0 - 10) were examined at the initiation of treatment and compared to their respective scores three months after beginning therapy. Toxicity was scored using the Common Terminology Criteria for Adverse Events (CTCAE) version 4.0. Statistical analyses and figure production was completed with the Statistical Package for the Social Sciences (SPSS) (IBM, Armonk, New York, USA) with a p < 0.05 considered significant. 

## Results

Patient, lesion, and treatment characteristics

Baseline patient characteristics are summarized in Table 1. A total of 45 rHNC patients treated with SBRT from 12 different radiotherapy centers in North America met the study criteria. Thirty-one patients in the cohort also had information regarding LC. Median follow-up was 8.78 months (range: 1 - 59.43 months).

**Table 1:** Summary of Patient Cohort Characteristics OS: overall survival; LC: local control; KPS: Karnofsky performance status; VAS: visual analogue scale

Variable	
Patients with data on OS	45 patients
Patients with data on LC	31 patients
Gender	
Male	29 patients (64.44%)
Female	16 patients (35.56%)
Median Age (range)	69 years (37 - 98)
Median Initial KPS (range)	80% (20% - 100%)
Median Initial VAS Pain Score (range)	3.50 (0 - 10)
Ethnicity	
Caucasian	34 patients (75.56%)
African American	4 patients (8.89%)
Asian	2 patients (4.44%)
Other	5 patients (11.11%)
Prior Treatments for Initial Lesion	
Surgery	18 patients (40.00%)
Chemotherapy	32 patients (71.11%)
Radiation Therapy	45 patients (100.00%)

Table 2 provides information regarding the characteristics of the rHNC lesions, radiotherapy dose, and LC and OS outcomes. The median GTV was 34.09 cc (range: 1.00 - 258.12 cc). One-third of the patients treated with SBRT had lesions involving the nasopharynx. Other common sites included the oropharynx, oral cavity, and tongue. The median prescription dose was 30 Gy (range: 20 - 42.5 Gy). 

**Table 2:** Summary of Lesion Characteristics and Radiotherapy Planning GTV: gross tumor volume; LC: local control; OS: overall survival; KPS: Karnofsky performance status; VAS: visual analogue scale; cc: cubic centimeters

Variable	
Median GTV (cc) (range)	34.09 (1.00 - 258.12)
Lesion Location	
Nasopharynx	15 patients (33.33%)
Gum, floor of mouth, other mouth	5 patients (11.11%)
Tongue	9 patients (20.00%)
Oropharynx	10 patients (22.22%)
Pharynx/location not specified	5 patients (11.11%)
Hypopharynx	1 patient (2.22%)
Median prescribed dose (range)	30 Gy (20 Gy - 42.5 Gy)
Median time to locoregional failure (LRF) (months) (range)	6.07 (1.5 - 59.43)
Median OS (months) (range)	9.23 (0.7 - 59.43)
Median KPS after treatment (range)	80% (10% - 100%)
Patients with rise in KPS	3 patients (14.29%)
Patients with no change in KPS	17 patients (80.95%)
Patients with decline in KPS	1 patient (4.76%)
Median VAS pain score after treatment (range)	0 (0 - 10)
Patients with rise in VAS pain score	3 patients (13.04%)
Patients with no change in VAS pain score	12 patients (52.17%)
Patients with decline in VAS pain score	8 patients (34.78%)

Following SBRT, the median time to locoregional failure (LRF) and the median OS were 6.07 months (range: 1.5 - 59.43 months) and 9.23 months (range: 0.7 - 59.43 months), respectively. One-year LC and OS rates for the cohort were 49.64% (95% confidence interval (CI) = 20.35% - 73.49%) and 37.76% (95% CI = 22.70% - 52.75%), respectively. Median KPS after treatment was unchanged at 80% (range: 10 - 100%). Fourteen percent, 81%, and 5% of the cohort had increases, stabilization, or declines in KPS following SBRT, respectively. Median VAS pain scores declined from 3.50 to zero, with 34.78% of the cohort reporting declines in pain, 52.17% of patients reporting stabilization of pain, and 13% of patients noting pain progression.

Overall survival

**Figure 1:**
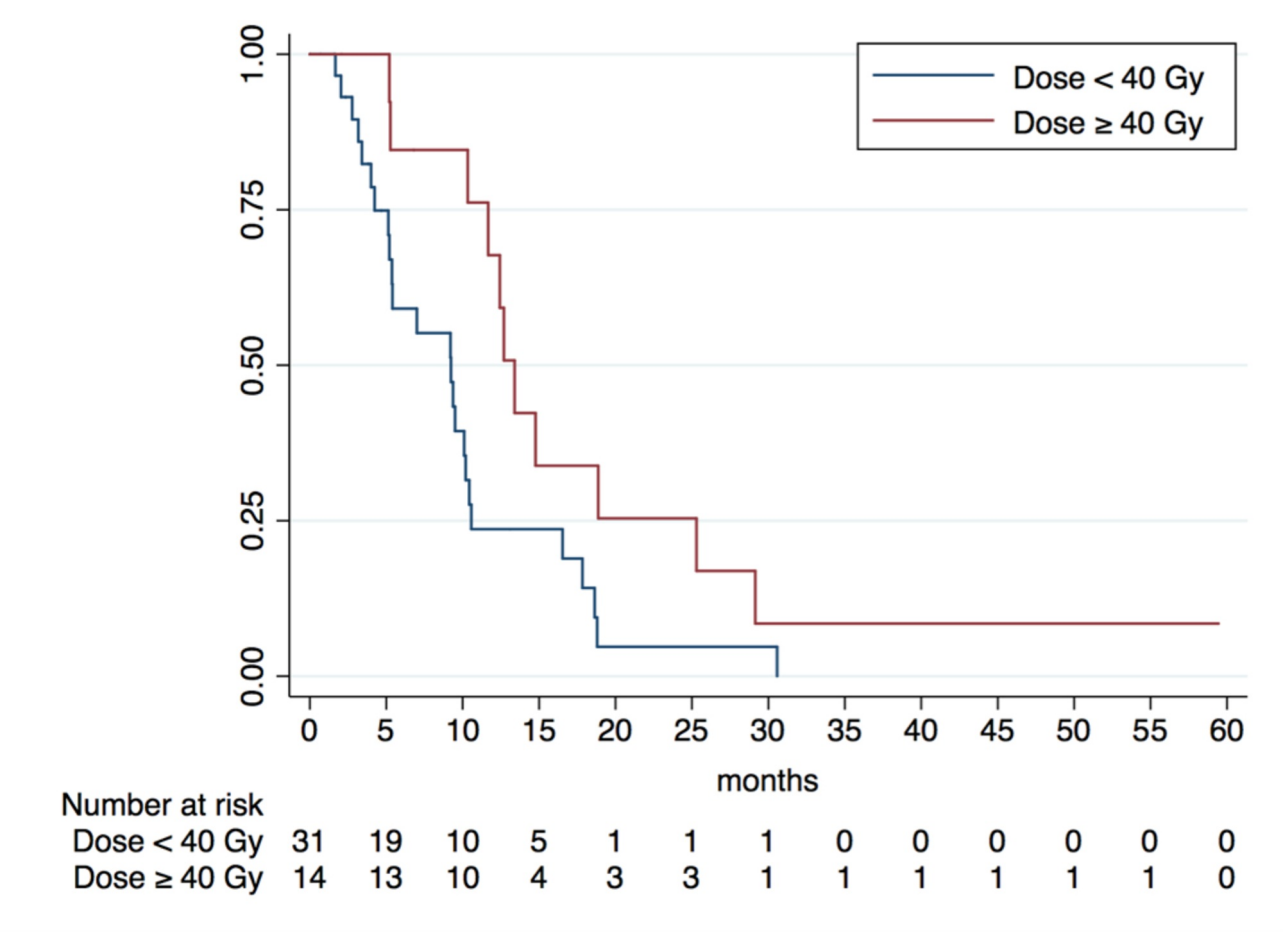
Kaplan-Meier Analysis of Overall Survival Benefit of Dose Escalation for Five-Fraction Stereotactic Body Radiotherapy

A summary of potential prognostic factors and their impact on OS is outlined in Table 3. While no significant difference was noted by factors (such as tumor volume, recurrence site, age, or KPS), patients treated with prescription doses ≥ 40 Gy had a significantly higher one-year OS of 67.79% (95% CI: 34.92% - 86.53%) versus 23.64% (95% CI: 9.66% - 41.07%) when compared with those receiving < 40 Gy (p = 0.028) (Figure 1). In order to correct for underlying balances due to retrospective design, an MVA was performed utilizing both forced entry and forward conditional selection methods. Both methods confirmed statistical significance with regards to dose escalation increasing OS (Table 3). On MVA using the forced entry model, prescription doses ≥ 40 Gy for five-fraction SBRT continued to be associated with significantly improved OS {hazard ratio (HR) = 0.45 (95% CI: 0.21 - 0.98; p = 0.045)} when controlling for tumor size, recurrence location, patient age, and patient KPS.

**Table 3:** Summary of Kaplan-Meier Analyses of Potential Prognostic Factors on One-Year Overall Survival OS: overall survival; CI: confidence interval; KPS: Karnofsky performance status; GTV: gross tumor volume; SBRT: stereotactic body radiation therapy; cc: cubic centimeters

Variable	Number of Patients	1-year OS (95% CI)	p-value
Initial KPS			0.315
< 80%	15 patients	35.71% (95% CI: 13.03 - 59.44%)	
≥ 80%	30 patients	39.25% (95% CI: 20.26 - 57.82%)	
Tumor Location			0.202
Other Locations	35 patients	42.66% (95% CI: 25.26 - 59.00%)	
Oropharynx	10 patients	15.43% (95% CI: 0.77 - 48.83%)	
Age			0.725
< 70 years	25 patients	38.79% (95% CI: 22.07 - 55.24%)	
≥ 70 years	20 patients	29.17% (95% CI: 4.23 - 61.88%)	
GTV			
< 25 cc	9 patients	25.93% (95% CI: 3.89 - 57.04%)	0.698
≥ 25 cc	36 patients	40.67% (95% CI: 23.31 - 57.35%)	
< 50 cc	14 patients	39.68% (95% CI: 14.78 - 63.96%)	0.598
≥ 50 cc	31 patients	36.71% (95% CI: 18.67 - 54.97%)	
Prescription Dose (Five-fraction SBRT)			0.028*
< 40 Gy	31 patients	23.64% (95% CI: 9.66 - 41.07%)	
≥ 40 Gy	14 patients	67.79% (95% CI: 34.92 - 86.53%)	

Local control

**Figure 2:**
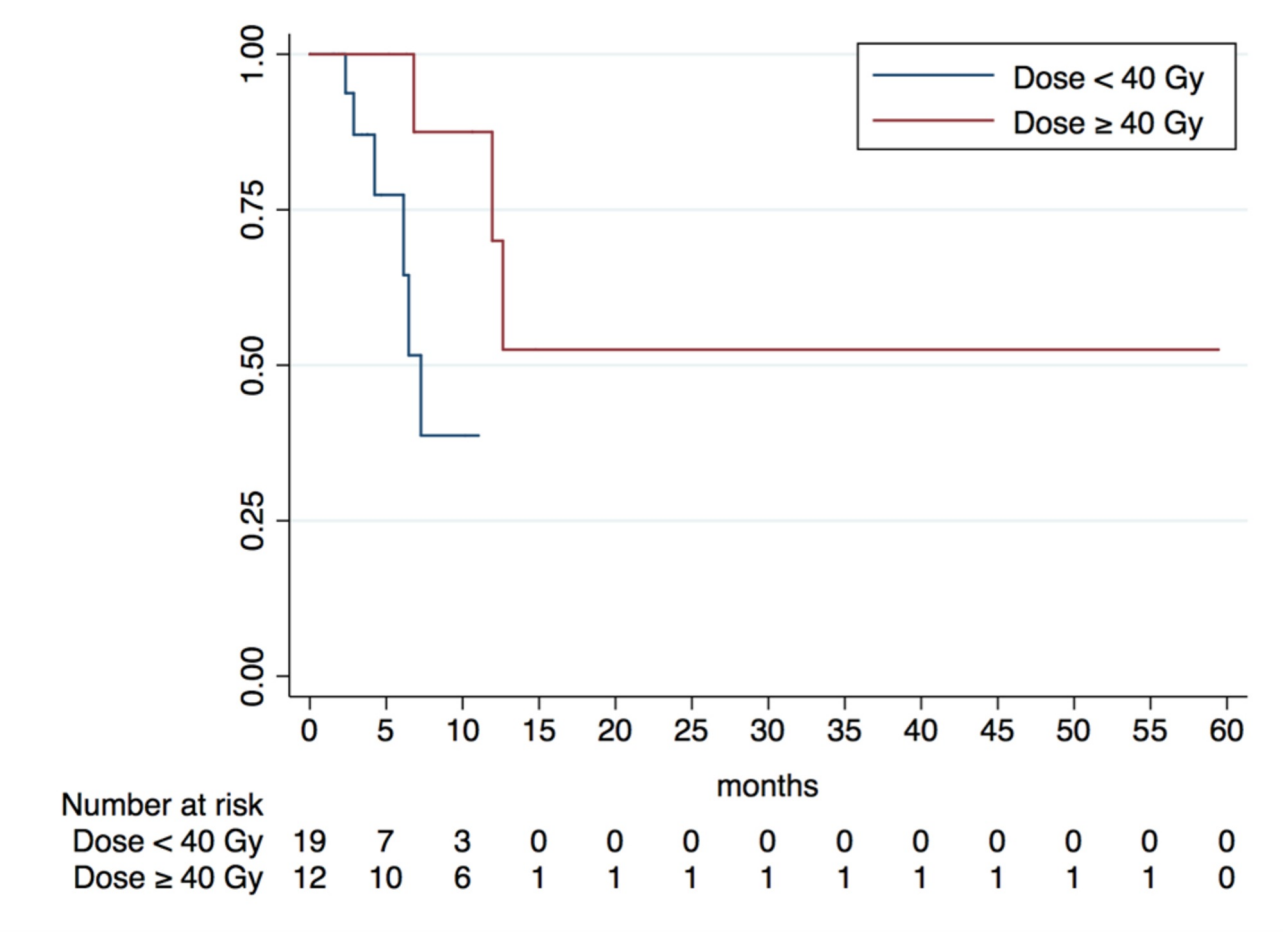
Kaplan-Meier Analysis of Local Control Benefit of Dose Escalation for Five-Fraction Stereotactic Body Radiotherapy

Table 4 depicts the impact of prognostic factors of interest on LC in our cohort. Consistent with findings regarding OS, patients treated to prescription doses ≥ 40 Gy resulted in significantly higher one-year LC rate of 75.00%, (95% CI: 22.48% - 91.83%) compared to the 36.84% one-year LC rate noted in those treated with < 40 Gy (95% CI: 9.67% - 67.98%, p = 0.030) (Figure 2). On MVA using forced entry analysis, prescription doses ≥ 40 Gy for five-fraction SBRTcontinued to be associated with significantly improved LC {HR = 0.086 (95% CI: 0.01 - 0.88); p = 0.038)}. Results were confirmed on sensitivity analysis with MVA formed using parsimonious forward selection methods.

**Table 4:** Summary of Kaplan-Meier Analyses of Potential Prognostic Factors on One-Year Local Control LC: local control; CI: confidence interval; KPS: Karnofsky performance status; GTV: gross tumor volume; SBRT: stereotactic body radiation therapy; cc: cubic centimeters

Variable	Number of Patients	1-year LC (95% CI)	p-value
Initial KPS			0.949
< 80%	12 patients	62.50% (95% CI: 22.93 - 86.07%)	
≥ 80%	19 patients	41.27% (95% CI: 7.55 - 73.85%)	
Tumor Location			0.175
Other Locations	24 patients	65.34% (95% CI: 34.90 - 84.20%)	
Oropharynx	7 patients	53.33% (95% CI: 6.83 - 86.31%)	
Age			0.379
< 70 years	16 patients	54.73% (95% CI: 20.38 - 79.58%)	
≥ 70 years	15 patients	46.67% (95% CI: 7.08 - 80.30%)	
GTV			0.792
< 25 cc	5 patients	47.39% (95% CI: 19.45 - 68.43%)	
≥ 25 cc	26 patients	51.84% (95% CI: 21.08 - 75.79%)	
< 50 cc	8 patients	27.78% (95% CI: 1.00% - 70.04%)	0.700
≥ 50 cc	23 patients	63.92% (95% CI: 32.59 - 83.64%)	
Prescription Dose (Five-fraction SBRT)			0.030*
< 40 Gy	19 patients	36.84% (95% CI: 9.67 - 67.98%)	
≥ 40 Gy	12 patients	75.00% (95% CI: 22.48 - 91.83%)	

Toxicities

Treatment-related side-effects (Table 5) were documented in 22.2% of all cases (10/45 patients) with 20% and 8.89% of patients reporting acute and late toxicities, respectively. The majority of patients reported CTCAE Grade 1 (66.6%) or Grade 2 (25.0%) toxicities for 11 unique side-effects among the 10 patients reporting treatment-related toxicities. Of the acute toxicities, 50% were of Grade 1 and 37.5% were of Grade 2, and there was one incidence of an acute Grade 3 esophagitis reported two months following completion of SBRT. All patient-reported late toxicities were of Grade 1. The most commonly reported side-effects were fatigue and dermatitis. Dose escalation was associated with a higher likelihood of treatment-related toxicities for patients treated and doses ≥ 40 Gy (33.33%) as compared to < 40 Gy (13.33%; p = 0.015).

**Table 5:** Summary of Patient-Reported Toxicities by Common Terminology Criteria for Adverse Events Grade

Grade 1 Toxicities	Grade 2 Toxicities	Grade 3 Toxicities
Fatigue (acute and late) - 3 patients	Fatigue (acute) - 1 patient	Esophagitis (acute) - 1 patient
Dermatitis (late) - 1 patient	Dermatitis (acute) - 1 patient	
Voice change (acute) - 1 patient	Skin ulceration (acute) - 1 patient	
Taste alteration (acute) - 1 patient		
Pain (acute and late) - 1 patient		
Dysphagia (late) - 1 patient		
Edema (late) - 1 patient		

## Discussion

Recurrence of HNC is a challenging clinical problem that often can lead to pain, suffering, and/or death [19]. Current salvage therapies (surgery, chemotherapy, immunotherapy, conventional re-irradiation) have limited efficacy and potentially significant toxicities [8, 15]. SBRT allows for more conformal delivery of tumoricidal doses of radiation and has the potential for superior LC, OS, and quality of life as compared to conventional RT [1].

On multivariate analysis, we found that patients treated with five-fraction SBRT who received doses ≥ 40 Gy had a significantly higher median OS (p = 0.028) and LC (p = 0.030) in comparison to those treated with < 40 Gy. These results are consistent with other studies demonstrating an association between LC and OS with higher prescription doses [1, 5, 20]. Rwigema et al. previously reported significantly improved three-year LC rates of 41.1% for patients treated with SBRT with prescription doses of 40 - 50 Gy as compared to 15.9% for those treated with less than 40 Gy [5]. Also, a recent multi-institutional analysis by Vargo et al. found that rHNC patients with GTVs ≤ 25 cc treated with SBRT of doses > 35 Gy had superior two-year OS rates compared to patients treated with doses < 35 Gy (38.5% vs. 14.4%) [1]. Our results also support tumor control probability data recently presented by the American Association of Physics in Medicine Working Group on Stereotactic Body Radiation Therapy (WGSBRT), which described a significant increase in LC and OS with dose escalation from 25 Gy to 40 Gy and recommended a minimum prescription dose of 40 Gy for all rHNC tumors treated with SBRT [21]. 

To examine the effect of GTV on OS and LC and compare to previous literature, we used cutoffs of 25 cc and 50 cc, which is consistent with other studies examining relationships between GTV and OS during the primary course of therapy [22-23]. Our investigation did not find a statistically significant relationship between GTV and OS, nor between GTV and LC. This can be compared to Rwigema et al., who found no significant correlation between GTV and OS but did note a significant improvement in LC based on GTV ≤ 25 cc [5].    

Among our cohort, the incidence of toxicity was relatively low, with 22.2% of patients reporting side effects. We found that 93.3% of these toxicities were Grade 1/2 with only one Grade 3 event of esophagitis occurring at two months post-treatment. These results are similar to the Phase I dose escalation study by Heron et al., which found no Grade 3/4 toxicities in a dose-escalation trial treating up to 44 Gy in five fractions over a two-week period [4]. Our findings are further supported in a study by Rwigema et al., which found dose escalation up to 50 Gy in five fractions to be feasible with no Grade 4/5 toxicities present [5]. However, our results did reveal a significant increase in toxicities following prescription doses ≥ 40 Gy for five-fraction SBRT (33.33%) as compared to < 40 Gy (13.33%; p = 0.015) that was not found by Rwigema et al. [5]. 

There are notable limitations to this study which merit attention. First, the smaller sample size resulting from our stringent selection criteria makes it challenging to generalize our findings to the general population. Second, data were censored for loss to follow-up during statistical analysis. Our study includes patients who were predominantly (89% of cohort) treated at community radiotherapy centers, which may contribute to differences in rates of follow-up and patient documentation compared to previous literature. Given the retrospective nature of the study, the lack of standardized follow-up limited the documentation of locoregional failures following SBRT and analyses examining LC. Additionally, while our rate of patient-reported toxicities is consistent with previous literature, the lack of homogenous follow-up increases the likelihood of under-reporting of toxicities. No information was available in the registry regarding initial disease staging, initial radiation therapy doses utilized, Epstein–Barr virus (EBV) status, human papillomavirus (HPV)/p16 staining on pathology, neoadjuvant, concurrent, or adjuvant chemotherapies or targeted therapies, the time between initial treatment and disease recurrence, or patient smoking status. Discrepancies in the diversity in our patient population are also notable, given that we have a higher than expected number of patients with nasopharyngeal disease despite few patients of Asian descent and a low number of African-American patients who normally bear a greater relative burden of HNC with poorer OS [24].

## Conclusions

In this analysis, we provide a unique method to analyze outcomes of rHNC patients treated with SBRT across numerous institutions. Our findings demonstrate the efficacy of utilizing SBRT in the treatment of rHNC, showing improved patient outcomes when using prescription doses ≥ 40 Gy with subsequent increases in toxicity. Recent research demonstrates that the use of SBRT in conjunction with epidermal growth factor receptor (EGFR) inhibitors, such as cetuximab, display promising potential. As such, follow-up studies and clinical trials should examine patient outcomes using SBRT to treat rHNC at doses ≥ 40 Gy in combination with novel therapeutic agents. Further research should also be directed at determining if the increases in toxicity from doses greater than 40 Gy lead to changes in patient quality of life. Finally, this study provides further evidence that multi-institutional, community-focused databases can be effective tools in providing accurate, comparable information on the outcomes of patients with rHNC treated with SBRT. Also, our research provides further support for the notion that SBRT to doses of ≥ 40 Gy can safely be applied to patients with rHNC across a variety of clinical practice settings. Given its capability to compare and contrast patient outcomes data across multiple academic and private-practice settings, we suggest future analyses using the RSSearch Patient Registry to examine outcomes of additional treatment sites as the database accrues more clinical information. 
